# Limbic system damage following SARS-CoV2 infection

**DOI:** 10.1093/braincomms/fcad340

**Published:** 2023-12-07

**Authors:** Aslihan Taskiran-Sag, Hare Yazgi

**Affiliations:** Department of Neurology, Faculty of Medicine, TOBB University of Economics and Technology, 06510, Ankara, Turkey; Faculty of Medicine, TOBB University of Economics and Technology, 06510 Ankara, Turkey

The World Health Organization declared end to coronavirus disease 2019 (COVID-19) pandemic on May 5, 2023. Certainly, this did not mean that SARS-CoV2 would not be a concern in our daily medical practice. As neurologists, we may even not have seen the major neurological disease burden caused by this virus regarding the long-term sequelae in COVID-19 survivors.

SARS-CoV2 is a neurotropic virus. Although direct evidence is not present, the virus is thought to enter the neural tissue either hematogenously or through peripheral nerve endings and access the brain via retrograde transport. The neurological symptoms of long COVID may arise from this invasion.^1^

There was a remarkable research article in *Brain Communications* in June 2023 by Thomasson *et al*. on limbic system damage following SARS-CoV2 infection.^2^ They delicately showed that moderately and severely affected COVID-19 patients displayed impaired emotion recognition which was also associated with decreased memory and olfactory abilities. Moreover, they identified altered functional connectivity patterns involving cortico-subcortical-cerebellar networks at 6–9 months post-infection.

From the very beginning, the limbic system has been under the spotlight since loss of olfaction was one of the major presenting symptoms of SARS-CoV2 infection. Subsequent studies have shown altered metabolism, perfusion, structure or connectivity in the limbic structures after COVID-19.^3-6^ This study adds to these data by showing the behavioural consequences of limbic system damage and associated functional connectivity changes simultaneously, 6–9 months after COVID-19. The main question we wonder about at this point is whether there were accompanying structural changes in the limbic sites of the brain. The authors tried to assess this issue by volume-based morphometric analyses. They reported no structural differences in the anatomical images that could be associated with the functional patterns. We would like to point out that the absence of a control group in the study makes it difficult to detect an attributable structural change. Alterations in limbic brain structures were evident even in mild, non-hospitalized patients in previous studies.^6,7^ Both atrophy and increase in thickness of cortical areas have been reported at varying time points after the infection—in the acute or subacute period.^8,9^ One of the main concerns relating SARS-CoV2 is secondary neurodegeneration and cerebral cortical atrophy is an important finding with this regard. In the long run, we may encounter function and network impairment without any structural damage (i.e. atrophy). However, if anatomical structure becomes atrophic along with the function and network disruptions, that can imply degenerative process and warrants special consideration. We think it may be useful to include this information in the valuable cohort of Thomasson *et al*. Additionally, Supplementary Table 4 in their article^2^ documents the authors’ volume-based morphometric findings and we could only see amygdala, but not hippocampus or insula, in the brain parcels of structural analyses. Insulae, hippocampus, parahippocampus, perirhinal and entorhinal cortices are frequently reported to be affected in long COVID.^6,7,10^ A detailed re-evaluation of these structures in the authors’ sample and comparison with an age- and sex-matched control group may yield important results in terms of neurodegenerative diseases anticipated after the pandemic.

In conclusion, SARS-CoV2 related neurological issues were not limited to the acute complications or long COVID and such research concerning the neural damage following SARS-CoV2 infection should continue unabated.

## Data Availability

Data sharing is not applicable to this article as no new data were created or analysed.
